# The STIM1/2-Regulated Calcium Homeostasis Is Impaired in Hippocampal Neurons of the 5xFAD Mouse Model of Alzheimer’s Disease

**DOI:** 10.3390/ijms232314810

**Published:** 2022-11-26

**Authors:** Ksenia Skobeleva, Alexey Shalygin, Elena Mikhaylova, Irina Guzhova, Maria Ryazantseva, Elena Kaznacheyeva

**Affiliations:** 1 Institute of Cytology of the Russian Academy of Sciences (RAS), 194064 St. Petersburg, Russia; 2 Neuroscience Center, University of Helsinki, HiLIFE, P.O. Box 63, 00014 Helsinki, Finland

**Keywords:** Alzheimer’s disease, 5xFAD, hippocampal cultures, store-operated calcium entry, voltage-gated calcium entry, STIM1, STIM2, leflunomide, nifedipine, BTP-2, calcium hypothesis

## Abstract

Alzheimer’s disease (AD) is the most common cause of age-related dementia. Neuronal calcium homeostasis impairment may contribute to AD. Here we demonstrated that voltage-gated calcium (VGC) entry and store-operated calcium (SOC) entry regulated by calcium sensors of intracellular calcium stores STIM proteins are affected in hippocampal neurons of the 5xFAD transgenic mouse model. We observed excessive SOC entry in 5xFAD mouse neurons, mediated by STIM1 and STIM2 proteins with increased STIM1 contribution. There were no significant changes in cytoplasmic calcium level, endoplasmic reticulum (ER) bulk calcium levels, or expression levels of STIM1 or STIM2 proteins. The potent inhibitor BTP-2 and the FDA-approved drug leflunomide reduced SOC entry in 5xFAD neurons. In turn, excessive voltage-gated calcium entry was sensitive to the inhibitor of L-type calcium channels nifedipine but not to the T-type channels inhibitor ML218. Interestingly, the depolarization-induced calcium entry mediated by VGC channels in 5xFAD neurons was dependent on STIM2 but not STIM1 protein in cells with replete Ca^2+^ stores. The result gives new evidence on the VGC channel modulation by STIM2. Overall, the data demonstrate the changes in calcium signaling of hippocampal neurons of the AD mouse model, which precede amyloid plaque accumulation or other signs of pathology manifestation.

## 1. Introduction

Alzheimer’s disease (AD) is the most common cause of age-related dementia. AD is a neurodegenerative disease that primarily affects hippocampal and cortical neurons, leading to memory loss and cognitive impairment. A familial form of AD (FAD), in most cases, is caused by mutations in *APP*, *PSEN1*, and *PSEN2* genes, manifests early and exhibits a severe phenotype (reviewed in [1]). Mouse models of FAD usually express mutant human *APP* and *PSEN1* genes associated with the disease. The 5xFAD transgenic mouse model is a double transgenic model with five FAD-associated mutations: Swedish (K670N, M671L), Florida (I716V), and London (V717I) mutations in the amyloid precursor protein (APP) gene, and M146L and L286V mutations in the presenilin-1 (*PSEN1*) gene under the control of the neuron-specific promoter Thy1 [2]. It has a phenotype with rapid amyloidogenesis, neuronal loss, behavior abnormalities, and memory deficits [2,3]. The amyloid plaques in the brain of 5xFAD mice manifest between 2 and 4 months of age. However, emerging evidence supports the idea that pathological changes related to FAD may occur earlier than amyloid plaque deposition [4,5]. Here, we use hippocampal neurons of 5xFAD mice cultured for 10–13 days in vitro. At this stage neurons do not exhibit severe amyloid pathology [6] but express human *APP* and *PSEN1* genes with mutations associated with familial AD [7].

A growing number of studies demonstrate deregulation of calcium homeostasis in modeling FAD and other neurodegenerative diseases in neurons of animals [3,8,9,10,11] (reviewed in [12,13,14,15]). Calcium homeostasis plays a critical role in neuron survival, synaptic transmission, and neural plasticity, as well as in the mitochondria function and cell metabolism (reviewed in [16,17]). Therefore, impairments in calcium homeostasis can affect many processes in neurons leading to neurodegeneration, cognitive impairment, and memory loss. There are two ways to raise calcium concentration within the neuron: by calcium entry through ionotropic receptors and ion channels in the plasmatic membrane (PM) and by calcium release from intracellular stores. Several publications have demonstrated changes in the endoplasmic reticulum (ER) calcium storage and release in neurons that appear earlier than the AD-associated plaques [18,19]. Calcium stores of the endoplasmic reticulum (ER) could rapidly release calcium to the cytosol through ryanodine- or IP3-calcium channels. Stromal interactional molecules (STIM) sense calcium concentrations within the ER and produce an intracellular signal in response to a drop in ER calcium [20]. STIMs are a central link between intracellular calcium stores and calcium channels in the plasma membrane (PM). They play a notable role in the regulation of calcium homeostasis in health and disease (reviewed in [21,22]). There are two homologs of STIMs, which differ in their role in calcium homeostasis and intracellular signaling. Both STIMs are known to activate store-operated calcium (SOC) entry by activating Orai and transient receptor potential canonical (TRPC) channels in the PM [23,24,25]. SOC entry disturbances were reported for 3xTg, PS1-M146V knock-in and APPKI transgenic mouse models of AD [26,27,28,29], and other neuronal models of AD and aging [26,27,30,31,32,33]. The STIM2 homolog has a lower affinity for calcium than STIM1 and, therefore, is more sensitive to small changes in ER luminal calcium concentrations. Tonic activation of STIM2 regulates basal calcium entry and refilling of ER stores, while STIM1 is regarded as a signal transducer of the more robust receptors-induced calcium release from ER [34,35]. STIM1 interacts with L-type and T-type voltage-gated calcium (VGC) channels and inhibits their activity [36,37,38]. Although STIM2 was described as a weaker CaV1.2 inhibitor in vascular smooth muscle cells, its role in VGC channel regulation has not been investigated further [37]. Moreover, despite the prominent STIM2 role in neuronal calcium homeostasis, it is unknown whether STIM2 regulates VGC channels in neurons. Considering the importance of SOC and VGC channels in neurons for processes such as synaptic plasticity and neurotransmission [39], the malfunction of STIMs may lead to drastic consequences for the brain. STIM1 expression was shown to be attenuated in the brains of patients with AD, possibly leading to hyperactivation of L-type VGC channels [40]. STIM2 functional deficiency was reported in the dendrites of neurons in mouse AD models [26,41]. Taking into account the role of STIM proteins in the modulation of VGC and SOC channels as well as ER store refilling, we aimed to address STIMs’ contribution to calcium homeostasis impairment in the hippocampal neurons of 5xFAD transgenic mice.

Here for the first time, we investigated functional roles of the STIMs in the disruption of calcium homeostasis in the cultured hippocampal neurons of the newborn 5xFAD transgenic mice. In particular, we addressed the contribution of STIMs to SOC and VGC entry by combining in vitro calcium imaging and viral-induced gene knock-down. In addition, we analyzed SOC and VGC entry sensitivity to the specific pharmacological inhibitors and FDA-approved drugs in vitro. 

## 2. Results

### 2.1. Store-Operated Calcium Entry Is Enhanced in 5xFAD Hippocampal Neurons

**Figure 1:**
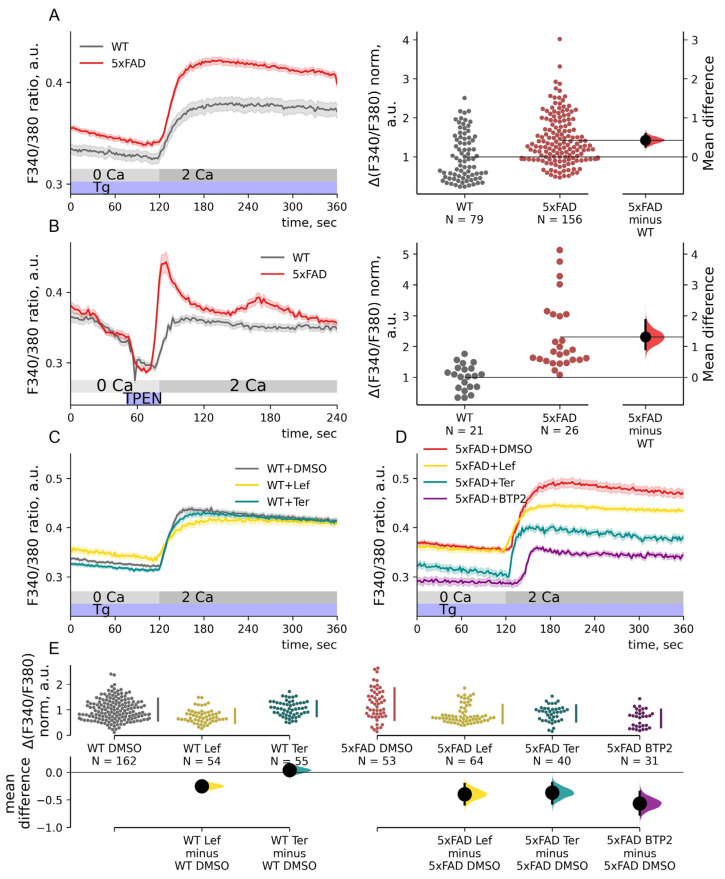
Effect of inhibitors Leflunomide, Teriflunomide, and BTP2 on elevated store-operated calcium entry in hippocampal neurons from 5XFAD mice. (**A**,**B**) Calcium imaging with Fura2 of neurons of primary hippocampal cultures of wild-type (WT, gray) or 5xFAD (red) mice. Mean traces of F340/380 ratio are represented as mean ± SEM. Calcium entry (ΔF340/F380) was calculated as the F340/F380 peak of each trace after Ca^2+^ add-back, and the baseline was subtracted. ΔF340/F380 data were normalized by the mean WT calcium entry and shown in the estimation plots. WT and 5xFAD calcium entry data are plotted on the left axes; the mean difference is plotted on floating axes on the right as a bootstrap sampling distribution. (**A**) Thapsigargin (Tg) was added to deplete internal calcium stores. The unpaired mean difference between WT and 5xFAD Tg-induced calcium entry is 0.42, *p* = 0.2 × 10^−6^, Mann–Whitney test. (**B**) Treatment with TPEN for 20 s led to Ca^2+^-binding and mimicking depleted ER stores. Ca^2+^ add-back resulted in calcium entry. The unpaired mean difference between WT and 5xFAD data is 1.31; *p* = 4.2 × 10^−7^, Mann–Whitney test. (**C**–**E**) Calcium imaging with Fura2 of primary hippocampal cell cultures of WT (**C**) or 5xFAD (**D**) mice treated for 20 h with 1 μM of leflunomide (Lef), teriflunomide (Ter), BTP2 or DMSO. 1 μM thapsigargin (Tg) was added to deplete internal calcium stores. Mean traces of F340/380 ratio are represented as mean ± SEM. Calcium entry (ΔF340/F380) was calculated as before and normalized by median calcium entry in WT neurons treated with DMSO and shown in the estimation plot. ΔF340/F380 data are plotted on the upper axes; mean differences are plotted on floating axes on the bottom as bootstrap sampling distributions. The Kruskal–Wallis ANOVA (*p* < 0.001) with post-hoc Dunn’s test was implemented; pairwise *p*-values were calculated. Mean differences between WT DMSO-treated neurons and WT neurons treated with Lef or Ter are −0.25 and 0.04; *p* = 0.002 and 1.0. Mean differences between 5xFAD DMSO-treated neurons and 5xFAD neurons treated with Lef, Ter or BTP2 are −0.40, −0.37 and −0.57; *p* = 2.4 × 10^−4^, 0.07 and 1.8 × 10^−6^.

Primary cultures of the hippocampi of 5xFAD mice and their non-transgenic littermates (WT) were probed with Fura2-AM to study store-operated calcium (SOC) entry. SOC entry in 5xFAD mice has not been addressed via calcium imaging before. We used thapsigargin (Tg), a sarco-/endoplasmic reticulum ATPase inhibitor, to deplete internal calcium stores. All solutions were supplemented with 1 μM TTX and 1 μM nifedipine to prevent neuronal activity. We measured SOC entry as calcium influx in 2 mM Ca^2+^ aCSF, which followed the internal calcium stores depletion with 1 μM Tg in a Ca-free solution. Calcium imaging revealed a 42% elevation of SOC entry in the 5xFAD hippocampal neurons compared to WT neurons (Figure 1A). Normalized SOC entry was 1.00 ± 0.07 for WT neurons and 1.42 ± 0.05 for 5xFAD neurons (*p* < 0.001, Mann–Whitney test). To study SOC entry without affecting store depletion/repletion machinery, we used chelator TPEN followed by Ca^2+^ add-back. TPEN chelates the calcium within internal stores, leading to SOC channel activation and calcium entry [42]. This approach confirmed that SOC calcium entry was elevated by 131%. Normalized calcium entry after TPEN treatment was 1.00 ± 0.09 for WT neurons and 2.31 ± 0.22 for 5xFAD neurons (*p* < 0.001, Mann–Whitney test, Figure 1B). 

### 2.2. FDA-Approved Drugs Reduce SOC Entry in 5xFAD Mouse Neurons

Inhibiting abnormal SOC entry could be a therapeutic opportunity to normalize calcium homeostasis. Screening of FDA-approved drug libraries yielded leflunomide and teriflunomide as possible SOC channel inhibitors [43]. The drugs have been approved for the treatment of inflammatory diseases such as arthritis and multiple sclerosis. Teriflunomide is a leflunomide metabolite that is converted in the plasma and the gastrointestinal tract [44]; both share structural similarities with a potent SOC channel inhibitor BTP2 [43]. Leflunomide and teriflunomide, as well as BTP2, demonstrated inhibition of SOC entry at clinically relevant doses. A 24 h pretreatment of rat basophilic leukemia cells with a low dose (1 μM) of a test drug reduced SOC entry [43]. Here we pretreated primary hippocampal cultures for 20 h with 1 μM leflunomide, teriflunomide, or 0.1% DMSO. The SOC entry was measured as a peak after Ca^2+^ add-back after 1 μM Tg treatment in Ca^2+^-free aCSF. Leflunomide reduced the SOC entry in both WT and 5xFAD neurons to 74% and 68% of the baseline compared to SOC entry in WT and 5xFAD neurons treated with DMSO (*p* < 0.001, Dunn’s test, Figure 1C–E). Normalized calcium entry for WT and 5xFAD neurons treated with DMSO were 1.00 ± 0.03 and 1.22 ± 0.08 and for WT and 5xFAD neurons treated with leflunomide, 0.74 ± 0.04 and 0.83 ± 0.05. Teriflunomide did not affect store-operated calcium entry in either WT or 5xFAD neurons: 1.04 ± 0.05 for WT and 0.85 ± 0.05 for 5xFAD (*p* > 0.5, Dunn’s test). BTP2 treatment reduced the store-operated calcium entry to 54% of the 5xFAD neurons baseline (0.66 ± 0.06; *p* < 0.001 compared to SOC entry in 5xFAD neurons treated with DMSO, Dunn’ test, Figure 1D,E). Therefore, SOC entry in 5xFAD hippocampal neurons was sensitive to inhibitors BTP2 and its FDA-approved analog leflunomide.

### 2.3. SOC Entry Enhancement in 5xFAD Mouse Neurons Is Associated with Changes in the Passive Leak but Not with Expression Levels of STIMs

The enhanced SOC entry in 5xFAD could be associated with ER store calcium levels, SOC machinery protein expression level, or SOC entry regulation. We addressed those possibilities to study the origin of the SOC entry increase. 

**Figure 2:**
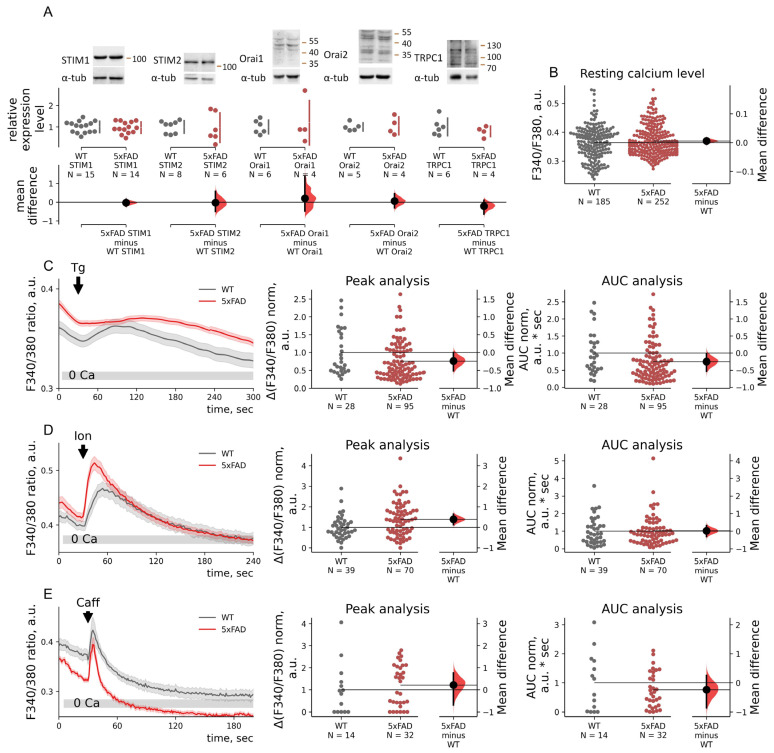
Intracellular basal calcium concentration or calcium store content and STIM, Orai, and TRPC1 protein expression levels are not affected in 5xFAD hippocampal neurons. (**A**) Western blots with neuronal culture lysates of wild-type (WT) and 5xFAD mice. Relative expression levels were calculated as densitometry of values for the target protein and normalized to α-tubulin values. Data are plotted on the upper axes; WT values were taken as 1; mean differences are plotted on floating axes on the bottom as bootstrap sampling distributions. Normalized data for WT and 5xFAD lysates were compared using unpaired *t*-test. Mean differences for STIM1 is −0.03 (*p* = 0.74), for STIM2 is (*p* = 0.90), for Orai1 is 0.20 (*p* = 0.67), for Orai2 is 0.05 (*p* = 0.80), and for TRPC1 is −0.21 (*p* = 0.38). (**B**) Resting calcium levels were measured as F340/F380 in neurons in 2Ca aCSF solution before any drugs application. Resting calcium levels in WT and 5xFAD neurons are shown in the estimation plot. Raw F340/F380 data are plotted on the left axes; the mean difference is plotted on floating axes on the right as a bootstrap sampling distribution. The mean difference between basal calcium entry of WT and 5xFAD neurons is 0.006; *p* = 0.75, Mann–Whitney test. (**C**–**E**) Calcium imaging with Fura2 of neurons of primary hippocampal cultures of wild-type (WT, gray) or 5xFAD (red) mice. Mean traces of F340/380 ratio are represented as mean ± SEM. Areas under the curve (AUC) are for response peaks after stimulation Ca^2+^ release. ΔF340/F380 and AUC values are normalized by the mean WT values and shown in estimation plots. ΔF340/F380 and AUC values are plotted on the left axes; the mean difference is plotted on floating axes on the right as a bootstrap sampling distribution. (**C**) 1 μM Thapsigargin (Tg) was added to deplete internal calcium stores. We excluded from analysis neurons that did not have Tg-induced calcium release. The normalized mean difference between Tg-induced Ca^2+^-release peaks of WT and 5xFAD neurons is −0.24; *p* = 0.04, Mann–Whitney test. The normalized mean difference between Tg-induced Ca^2+^-release AUC values of WT and 5xFAD neurons is −0.25; *p* = 0.03, Mann–Whitney test. (**D**) Five μM ionomycin (Ion) was added to deplete internal calcium stores. The normalized mean difference between Tg-induced Ca^2+^-release peaks of WT and 5xFAD neurons is 0.39; *p* = 0.009, Mann–Whitney test. The normalized mean difference between Tg-induced Ca^2+^-release AUC values of WT and 5xFAD neurons was 0.02; *p* = 0.08, Mann–Whitney test. (**E**) An amount of 25 mM caffeine (Caff) was added to evoke caffeine-induced calcium release driven by RyRs. The normalized mean difference between Tg-induced Ca^2+^-release peaks of WT and 5xFAD neurons is 0.22; *p* = 0.28, Mann–Whitney test. The normalized mean difference between Tg-induced Ca^2+^-release AUC values of WT and 5xFAD neurons is −0.24; *p* = 0.84, Mann–Whitney test.

The resting calcium levels measured in 2 mM Ca^2+^ aCSF at the beginning of experiments were similar between 5xFAD and WT neurons (Figure 2B). On the other hand, Tg-induced calcium release was significantly lower in the 5xFAD mouse neurons in respect to both amplitude and total calcium release assessed as an area under the curve (AUC). The reduction was estimated at about 25% (−0.24 for amplitude peaks and −0.25 for their AUC; *p* = 0.04 and 0.03, Mann–Whitney test). Moreover, we found a difference in the stores’ sensitivity to Tg as 5xFAD mouse neurons more frequently responded with calcium release. Tg failed to induce calcium release in 65% (*n* = 51/79) and 39% (*n* = 61/156) in WT and 5xFAD neurons correspondingly; Fisher’s exact conditional *p*-value = 0.3E-4. Application of 5 μM ionomycin in Ca^2+^-free aCSF led to calcium release in both 5xFAD and WT neurons (Figure 2D), showing no statistically significant difference in total intracellular Ca^2+^ store content assessed as an area under the curve (AUC); *p* = 0.84, Mann–Whitney test. Caffeine-induced calcium release, driven by RyRs activation, was similar in its amplitude, AUC, and slope in 5xFAD and WT neurons (Figure 2E), showing the absence of impact on the caffeine-sensitive/RyR-regulated stores; *p* = 0.28 for amplitude and 0.84 for AUC, Mann–Whitney test. We analyzed protein levels of key SOC entry molecular players in hippocampal neurons: channel subunits Orai1, Orai2, TRPC1, and calcium sensors STIM1 and STIM2 [45,46,47]. We found no difference in the protein levels in total lysates of WT and 5xFAD hippocampal cultures (*p* > 0.05, *t*-test, Figure 2A). Immunofluorescence of neurons stained with STIM1 or STIM2 antibodies did not reveal any difference in STIM1 or STIM2 protein levels between WT and 5xFAD neurons (Supplementary Figure S1).

### 2.4. STIM1 Contribution to SOC Entry Is Increased in 5xFAD Mouse Neurons

**Figure 3:**
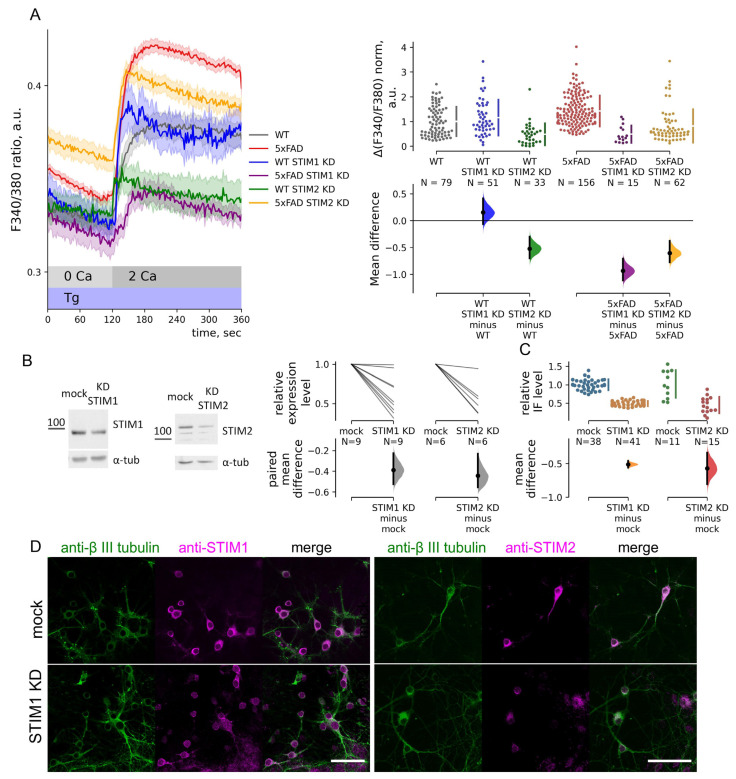
Store-operated calcium entry regulation by STIM1/2 proteins is changed in 5xFAD hippocampal neurons. (**A**) Calcium imaging with Fura2 of neurons of primary hippocampal cultures of wild-type (WT, gray) or 5xFAD (red) mice and treated with STIM1/STIM2 shRNA lentiviruses (STIM1 KD and STIM2 KD) or mock shRNA lentivirus. Mean traces of F340/380 ratio are represented as mean ± SEM. Thapsigargin (Tg) was added to deplete internal calcium stores. Calcium entry (ΔF340/F380) was calculated as the F340/F380 peak of each trace after Ca^2+^ add-back, and the baseline was subtracted. ΔF340/F380 data are normalized by the mean WT calcium entry and shown in the estimation plot. ΔF340/F380 data are plotted on the upper axes; mean differences are plotted on floating axes on the bottom as bootstrap sampling distributions. The Kruskal–Wallis ANOVA (*p* < 0.0001) with post-hoc Dunn’s test was implemented; pairwise *p*-values were calculated. Mean differences between calcium entry in WT and WT STIM1 KD or WT STIM2 KD neurons are 0.15 and −0.52; *p* = 1.0 and 2.3 × 10^−4^ compared to WT neurons. The mean difference between calcium entry in 5xFAD and 5xFAD STIM1 KD or 5xFAD STIM2 KD neurons are −0.94 and −0.61; *p* = 1.8 × 10^−7^ and 6.4 × 10^−11^ compared to 5xFAD. (**B**) Western blots with neuronal culture lysates. Neurons were treated with STIM1/STIM2 shRNA lentiviruses (STIM1 KD and STIM2 KD) or mock shRNA lentivirus (mock). Relative expression levels were computed as STIM1/2 densitometry values and normalized to α-tubulin values. Data pairs are plotted on the upper axes; mock values were taken as 1; mean differences are plotted on floating axes on the bottom as a bootstrap sampling distribution. The paired mean difference between mock and STIM1 KD or STIM2 KD lysates are −0.39 and −0.44 (*p* = 0.001 and 0.003, paired *t*-test). (**C**,**D**) Confocal immunofluorescence images of neuronal cultures probed with anti-STIM1/2 (magenta, anti-STIM1 and anti-STIM2) and β-III neuro-tubulin antibodies (green, anti-β III neuro-tubulin). Scale bar is 50 μm. Integrated immunofluorescence for anti-STIM1/2 staining was calculated for every neuron counterstained with anti-β III neuro-tubulin. Datasets were normalized to mock shRNA lentivirus values (mock) to determine relative immunofluorescence (IF) levels. Normalized data are plotted on the upper axes; mean differences are plotted on floating axes on the bottom as a bootstrap sampling distribution. Mean differences in relative immunofluorescence levels between mock and STIM1 KD or STIM2 KD neurons are −0.51 and −0.52 (*p* < 0.001, *t*-test).

To determine the role of regulation by STIMs in the excessive SOC entry, we performed a knock-down of STIM1 and STIM2 with shRNAs (STIM1 KD and STIM2 KD). STIM1 and STIM2 protein levels were reduced by the knock-down approximately to 40–50% of the baseline (mock shRNA) (Figure 3B,C). Calcium imaging revealed that neurons of wild-type mice with STIM1 KD had the same level of SOC entry as mock shRNA controls (Figure 3A). Normalized Tg-induced calcium entry amplitudes (ΔF340/F380) were 1.00 ± 0.07 for WT neurons and 1.15 ± 0.10 for WT STIM1 KD neurons (*p* = 1, Dunns’ test). On the other hand, the STIM1 KD in 5xFAD neurons decreased SOC entry by 66%, leading to a lower SOC entry than in WT neurons. Normalized Tg-induced calcium entry amplitudes (ΔF340/F380) were 1.42 ± 0.05 for 5xFAD neurons and 0.49 ± 0.09 for 5xFAD STIM1 KD neurons (*p* < 0.001, Dunns’ test, Figure 3A). Meanwhile, STIM2 KD decreased SOC entry in both WT and 5xFAD neurons to comparable levels. ΔF340/F380 normalized data for WT STIM2 and 5xFAD STIM2 were 0.48 ± 0.08 and 0.82 ± 0.09, and *p*-values of the Dunns’ test were <0.001 compared to WT and 5xFAD neurons (Figure 3A). The data indicates a dominant role of STIM1 contribution to SOC entry in 5xFAD hippocampal neurons. 

### 2.5. Depolarization-Induced Calcium Entry Is Enhanced in 5xFAD Neurons

**Figure 4:**
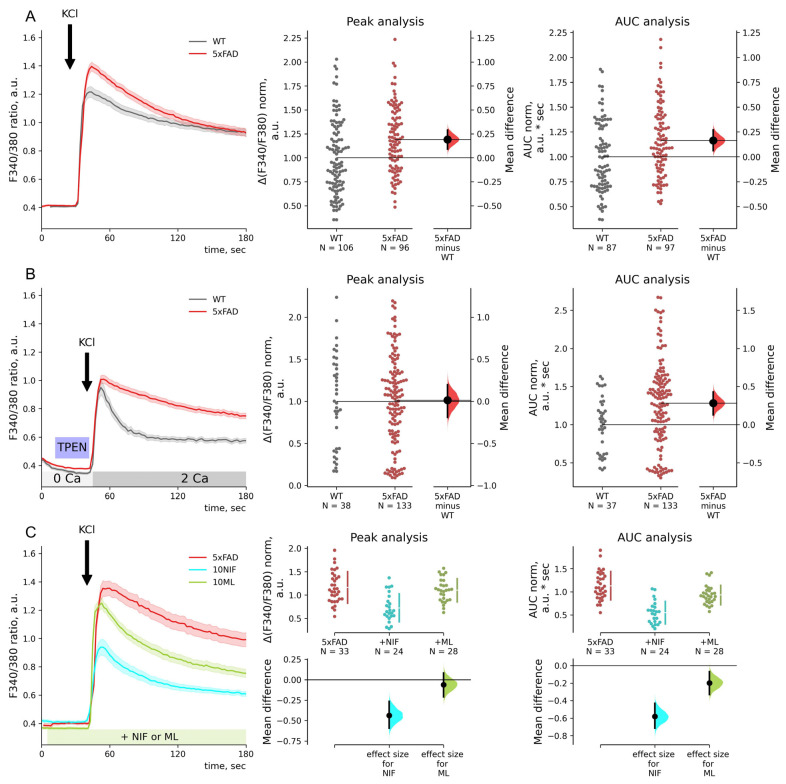
In hippocampal neurons, voltage-gated calcium entry is sensitive to intracellular calcium stores. (**A**–**C**) Calcium imaging with Fura2 of neurons of primary hippocampal cultures of wild-type (WT, gray) and 5xFAD mice. Mean traces of F340/380 ratio are represented as mean ± SEM. 50 mM KCl (KCl) was added to induce depolarization. Calcium entry (ΔF340/F380) was calculated as the F340/F380 peak of each trace after KCl application, and the baseline was subtracted. Areas under the curve (AUC) were calculated for 40 s after KCl applications. ΔF340/F380 and AUC data are normalized by the mean WT neuron values and shown in separate estimation plots. The estimation plots have normalized data plotted on the upper axes and mean differences plotted on floating axes on the bottom as a bootstrap sampling distribution. (**A**) The mean difference between KCl-induced calcium entry in WT and 5xFAD neurons is 0.19 for amplitudes and 0.17 for AUC values; *p* = 1.6 × 10^−4^ and 0.002, Mann–Whitney test. (**B**) Calcium stores were depleted with 1 mM chelator TPEN for 20 s before KCl application. The mean difference between KCl-induced calcium entry in WT and 5xFAD neurons is 0.01 for amplitudes and 0.28 for AUC values; *p* = 0.98 and 0.006, Mann–Whitney test. (**C**) Inhibitory analysis of depolarization-induced calcium entry in 5xFAD neurons was done with the Kruskal–Wallis ANOVA (*p* < 0.001 for peaks and AUC values) with post hoc Dunn’s test was implemented; pairwise *p*-values were calculated for untreated 5xFAD neurons. Mean differences between KCl-induced calcium entry in 5xFAD neurons and 10 μM nifedipine or ML218-treated 5xFAD neurons are −0.44 and −0.06 (*p* = 3.2 × 10^−5^ and 1.0) for amplitudes and −0.58 and −0.20 (*p* = 1.9 × 10^−9^ and 0.10) for AUC values.

To address the effect of endogenous STIMs on VGC entry in 5xFAD mice hippocampal neurons, we induced depolarization of the cells by applying high K+ aCSF (50 mM KCl). Depolarization-induced calcium entry was elevated by 20% compared to WT (1.00 ± 0.04 and 1.19 ± 0.05, *p* < 0.001 Mann–Whitney test, Figure 4A). Calcium stores depletion with 1 mM chelator TPEN reduced amplitudes of KCl-induced calcium entry to the same level in WT and 5xFAD neurons. The store depletion reduced VGC entry in both groups of neurons, with the 5xFAD mouse neurons decreasing the AUC of the response peak by 41% compared to the replete state (0.93 ± 0.04, *p* < 0.001, Dunn’s test), while WT mice show a reduction by 27% compared to WT replete state (0.73 ± 0.04, *p* = 0.036, Dunn’s test). These data pointed out that the increase in VGC entry in 5xFAD neurons is due to the store-sensitive VGC component. At the same time, the total calcium entry after the stores depletion, assessed as AUC, remained significantly elevated in 5xFAD neurons (1.00 ± 0.02 for WT and 1.28 ± 0.05 for 5xFAD; *p* = 0.006, Mann–Whitney test, Figure 4B). Therefore, the part of the difference in VGC entry was not dependent on ER calcium release or ER calcium content and persisted in the store-depleted condition. 

The excessive VGC entry in 5xFAD neurons was mainly sensitive to the L-type VGC channel inhibitor nifedipine (10 μM) but not to the T-type channel inhibitor ML218 (Figure 4C). Nifedipine reduced amplitude by 37% in 5xFAD neurons (0.73 ± 0.06 *p* < 0.001, Dunn’s test). KCl-induced calcium entry in control neurons was sensitive to both L- and T-type VGC channel inhibitors. Nifedipine reduced calcium entry amplitude by 26% (0.74 ± 0.04, *p* < 0.001, Dunn’s test), and ML218 by 15% (0.85 ± 0.04, *p* = 0.01, Dunn’s test).

**Figure 5:**
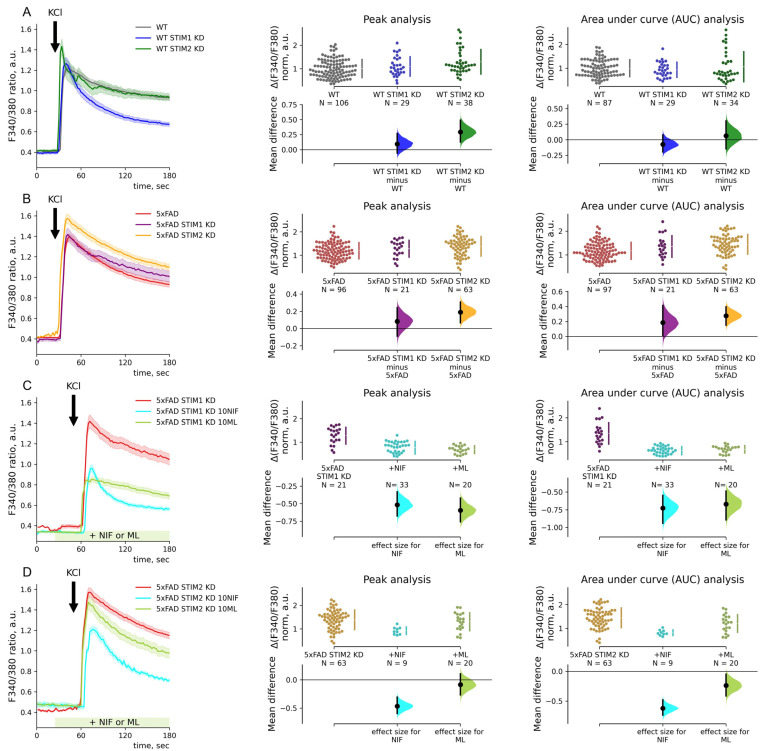
In hippocampal neurons, voltage-gated calcium entry is regulated by STIM2 sensors. (**A**–**D**) Calcium imaging with Fura2 of neurons of primary hippocampal cultures of wild-type (WT, gray) and 5xFAD mice and treated with STIM1/STIM2 shRNA lentiviruses (STIM1 KD and STIM2 KD) or mock shRNA lentivirus. Inhibitory analysis has been performed with 10 μM nifedipine (NIF), an inhibitor of L-type calcium channels, and 10 μM ML218 (ML), an inhibitor of T-type calcium channels. The inhibitors were introduced 1 min before KCl application. Mean traces of F340/380 ratio are represented as mean ± SEM. 50 mM KCl (KCl) was added to induce calcium entry. Calcium entry (ΔF340/F380) was calculated as the F340/F380 peak of each trace after KCl application, and the baseline was subtracted. Areas under the curve (AUC) were calculated for 40 s after KCl applications. ΔF340/F380 and AUC data are normalized by the mean WT neuron values and shown in separate estimation plots. The estimation plots have normalized ΔF340/F380 data plotted on the upper axes and mean differences plotted on floating axes on the bottom as bootstrap sampling distributions. **(A)** The Kruskal–Wallis ANOVA (*p* < 0.01 for peaks and AUC values) with post hoc Dunn’s test was implemented; pairwise *p*-values were calculated for WT values comparison. Mean differences between KCl-induced calcium entry in WT and WT STIM1 KD or WT STIM2 KD neurons are 0.09 and 0.29 with *p* = 0.91 and 0.005 for amplitudes and −0.07 and 0.06 with both *p* = 1.0 for AUC values. (**B**) The Kruskal–Wallis ANOVA (*p* < 0.001 for peaks and AUC values) with post hoc Dunn’s test was implemented; pairwise *p*-values were calculated for 5XFAD values comparison. Mean differences between KCl-induced calcium entry in 5xFAD and 5xFAD STIM1 KD or 5xFAD STIM2 KD neurons are 0.08 and 0.19 (*p* = 0.78 and 0.005) for amplitudes and 0.18 and 0.28 (*p* = 0.26 and 4.8 × 10^−5^) for AUC. (**C**) The Kruskal–Wallis ANOVA (*p* < 0.001 for peaks and AUC values) with post hoc Dunn’s test was implemented; pairwise *p*-values were calculated for 5xFAD neurons with STIM1 KD comparison. Mean differences between KCl-induced calcium entry in 5xFAD STIM1 KD neurons and 10 μM nifedipine or ML218 treated 5xFAD STIM1 KD neurons are −0.52 and −0.60 (*p* = 4.4 × 10^−5^ and 0.3 × 10^−5^) for amplitudes and −0.73 and −0.67 (*p* = 6.6 × 10^−9^ and 2.1 × 10^−5^) for AUC values. (**D**) The Kruskal–Wallis ANOVA (*p* < 0.004 for peaks and AUC values) with post hoc Dunn’s test was implemented; pairwise *p*-values were calculated for 5xFAD neurons with STIM2 KD comparison. Mean differences between KCl-induced calcium entry in 5xFAD STIM2 KD neurons and 10 μM nifedipine or ML218 treated 5xFAD STIM2 KD neurons are −0.47 and −0.08 with *p* = 0.002 and 1.0 for amplitudes and −0.62 and −0.24 with *p* = 8.2 × 10^−5^ and 0.09 for AUC values.

STIM1 KD did not impact the KCl-induced calcium entry peak amplitudes in hippocampal neurons from WT or 5xFAD animals. Normalized calcium entry amplitudes were 1.09 ± 0.08 for WT STIM1 KD neurons and 1.28 ± 0.08 for 5xFAD STIM1 KD neurons, *p* > 0.5, Dunn’s test (Figure 5A,B). However, STIM2 KD enhanced calcium entry in both WT and 5xFAD neurons (Figure 5A,B). Furthermore, in neurons from 5xFAD mice, STIM2 KD induced both higher peaks and long-lasting elevation of intracellular calcium increase after depolarization. Normalized calcium entry amplitude was 1.38 ± 0.05 (*p* = 0.005, Dunn’s test), and AUC value was 1.44 ± 0.05 (*p* < 0.001, Dunn’s test). The data imply that STIM2 regulates depolarization-induced VGC entry in hippocampal neurons with replete stores.

Experiments with pharmacological inhibitors of the VGC channels demonstrated that around 50% of KCl-induced calcium entry amplitude was sensitive to an L-type calcium channel inhibitor (10 μM nifedipine) in 5xFAD neurons with STIM1 or STIM2 KD, which was not different from neurons with mock shRNA or untreated 5xFAD neurons (the Kruskal–Wallis ANOVA is *p* > 0.05). The nifedipine reduced the calcium entry amplitudes by 41% in 5xFAD with STIM1 KD neurons (0.76 ± 0.05, *p* <0.001, Dunn’s test, Figure 5C) and by 33% in 5xFAD neurons with STIM2 KD (0.92 ± 0.05, *p* < 0.001, Dunn’s test, Figure 5D). Surprisingly, the STIM1 KD in 5xFAD neurons affected the sensitivity of VGC entry to the inhibitor of T-type calcium channels (10 μM ML218), leading to VGC entry being blocked by the inhibitor by 46% compared to 5xFAD mock-treated neurons (0.68 ± 0.04, *p* < 0.001, Dunn’s test). The STIM2 KD or mock shRNA did not affect the sensitivity of VGC entry to ML-218; amplitudes were 1.10 ± 0.09 for mock shRNA and 1.30 ± 0.08 for STIM2 KD 5xFAD neurons; *p* > 0.05, Dunn’s test, Figure 5C,D.

## 3. Discussion

### 3.1. STIM1 Drives Increased SOC Entry in Hippocampal Neurons of the 5xFAD Mouse Model

Several studies have demonstrated diverse effects on SOC entry in neurons of AD animal models, AD patient cells, and other cellular models (reviewed in [12,48]). A variety of mechanisms have been proposed to explain those effects: changes in expression levels of STIM proteins [26,40,49], changes in ER calcium storage and/or release [8,10,50,51], and enzymatic cleavage of STIM1 by gamma-secretase [52]. We studied early cultures (10–13 day in vitro) of the hippocampus of 5xFAD mice with human *APP* and *PSEN1* transgenes driven by a neuron-specific Thy1 promoter. Thy1 drives expression of transgenes in the hippocampus of E17.5 mouse embryos [53] and in primary cultures of neurons as early as 4–5 days in vitro. Cultured hippocampal 5xFAD neurons at 10–13 days in vitro exhibit neither severe amyloid pathology, due to limited time to accumulate human APP and PS1 transgenes, nor detectable level of amyloids [6]. Meanwhile, changes in calcium signaling were demonstrated already at this stage in cultured neurons of mouse transgenic AD models such as 3xTg and PS2(N141I) [27,54]. Here we observed for the first time elevated SOC entry in the neurons of 10–13 day in vitro primary cultures from the hippocampus of 5xFAD newborn mice. Both STIM homologs were involved in the elevated SOC entry. At the same time, the contribution of STIM1 to the somatic SOC entry in 5xFAD hippocampal neurons was more pronounced compared to neurons of WT mice. The effect was not associated with changes in protein levels of STIM1, STIM2, Orai1, Orai2, and TRPC1. The ER store content, estimated as ionomycin (by AUC) or caffeine-induced calcium release, was not different between 5xFAD and WT hippocampal neurons. At the same time, Tg-induced calcium release was decreased in the 5xFAD neurons, while neurons were more readily responsive to the Tg application. Therefore, the difference in SOC entry was not caused by changes in the bulk calcium in the ER stores or by changes in the levels of key SOC entry proteins. We speculate that ER stores in the 5xFAD mouse hippocampus have a higher sensitivity for Tg-induced calcium release, which increases the contribution of STIM1 in SOC entry regulation. This is in line with previous studies [31,55,56,57] where the passive leak, enhanced calcium store release, or STIM1 contributed to the disruption of calcium homeostasis in the neurons of AD models. The contribution of STIM1 and STIM2 splice isoforms is yet to be investigated [58,59]. 

Previously, we have demonstrated elevation of somatic STIM1-induced SOC entry in mouse hippocampal neurons expressing human presenilin-1 ΔE9 associated with FAD [31]. Later the enhancement was confirmed in dendritic spines of presenilin-1 ΔE9 expressing neurons [33]. However, several other AD models, including 3xTg, PS1-M146V knock-in and APP knock-in transgenic mice, demonstrated diminished SOC entry in soma and dendrites of neurons [26,27,29,41]. The difference in SOC entry between AD models could be an effect of different AD pathological mutations and variations in somatic/dendritic measurements. Pharmacological modulation of the SOC entry has been proposed as a strategy for treating neurodegenerative diseases (reviewed in [12,48]). For example, SOC entry inhibitor EVP4593 was shown to restore mushroom spines in mouse hippocampal neurons expressing presenilin-1 ΔE9 [33]. A less specific SOC entry inhibitor 2-APB was able to restore short-term memory in the Drosophila model of FAD [31] and restore LTP in the hippocampus of the FAD mouse model expressing presenilin-1 ΔE9 [60]. Here we demonstrated that elevated SOC entry in 5xFAD mouse hippocampal neurons was sensitive to the FDA-approved immunomodulatory drug leflunomide. The drug has been studied as an inhibitor of Orai1 and TRPC-dependent SOC entry at clinically relevant doses but has never been tested in neuronal cultures [43]. Pharmacological data can be useful for the future understanding of the drug’s action and side effects. 

### 3.2. STIM2 Regulates VGC Entry in 5xFAD Neurons with Replete ER Calcium Store

Several studies suggested the contribution of VGC channels in the pathology of AD based on post-mortem samples and animal models [61,62,63,64,65]. Here we observed the elevation of VGC entry in hippocampal neurons of 5xFAD mice in response to depolarization with 50 mM KCl. Increased VGC entry was sensitive to L-type channels inhibitor nifedipine but not to T-type channels inhibitor ML-218. The data is in line with previously observed elevated VGC entry in 3xTG and cellular AD models [40,63]. Both store-sensitive and insensitive VGC channels (defined by the sensitivity to store depletion with TPEN) contributed to the elevated calcium entry after depolarization. 

It has been reported earlier that STIM1 acts as a negative regulator of endogenous or overexpressed L-type calcium channels upon ER calcium store depletion in arterial smooth muscle cells (A7r5 VSMCs), cortical neurons, Jurkat T cells, HEK293 cells, and neuroblastoma Neuro2a [36,37]. The same effect upon SR store depletion has been reported for the endogenous T-type calcium channels in HL-1 cells [38]. The purported mechanism involves a reciprocal regulation of SOC and VGC channels by STIM1 upon store depletion and receptor-induced calcium release [36,37,66]. Although the STIM1 KD did not affect the VGC entry in VSMCs at a steady state (without store depletion), the STIM1-KO was reported to increase VGC entry in differentiated human neuroblastoma SH-SY5Y under the same conditions [40,67]. In contrast to STIM1, its ortholog STIM2 was described as a weaker negative regulator of voltage-gated calcium channels when it was overexpressed in VSMCs [37]. STIM2 KD in a VSMC line and human iPSC-derived GABAergic neurons did not affect the VGC entry with replete ER stores [67,68]. Here we studied endogenous VGC channel activation in hippocampal neurons without internal calcium stores pre-depletion. Our data demonstrated an absence of the effect of STIM1 KD on the VGC entry induced by depolarization with 50 mM KCl. At the same time, we observed an increase in VGC entry due to STIM2 KD. STIM2 is known to regulate steady-state calcium entry and could be active in resting neurons, as it was shown for hippocampal and cortical neuronal cultures [34,35,69]. The influence of overexpressed STIM1 on VGC entry was increased by STIM1 association with the plasma membrane. STIM2 proteins have a more robust association with the plasma membrane under resting conditions [70], which probably facilitates VGC channel regulation by STIM2. Interestingly, depolarization-induced calcium entry in neurons with STIM1 KD is more sensitive to ML-218 inhibitors of T-type VGC channels. Further investigation is required to reveal the mechanisms of VGC channel regulation by STIMs. 

Overall, our data demonstrate the changes in calcium signaling of hippocampal neurons of the AD mouse model, which precede amyloid plaque accumulation or other signs of pathology manifestation. Activity of both store-operated and voltage-gated calcium channels were affected in 5xFAD mouse neurons. Excessive store-operated calcium entry in 5xFAD is mediated by STIM1 and is sensitive to the FDA-approved drug leflunomide. Moreover, for the first time, we showed that STIM2 regulates voltage-gated calcium entry in hippocampal neurons. The data obtained could help to develop a strategy for treatment at the early stage of AD.

## 4. Materials and Methods

### 4.1. Animals

Hemizygous 5xFAD mice, their non-transgenic littermates, and C3HA mice of both sexes were used. Non-transgenic littermates were taken as controls and referred to as wild-type (WT). The 5xFAD transgenic mouse model is a double transgenic model with five FAD-associated mutations: Swedish (K670N, M671L), Florida (I716V), and London (V717I) mutations in the *APP* gene, and M146L and L286V mutations in the *PSEN1* gene under the neuron-specific Thy1 promoter [2]. Transgenic mice were acquired from the Jackson Laboratory and maintained on a mixed SJL/C57Bl6. All animal research was carried out under the guidelines for the welfare of animals of the ethical committee of the Institute of Cytology, Russian Academy of Sciences (No. F18-00380, approved on 12 October 2017). The animals were maintained in their home cages in a climate-controlled room (21–23 °C) with a 12:12 h light–dark cycle and had free access to water and food. 5xFAD mice were genotyped according to Jackson Laboratory protocol (№31769) with polymerase chain reaction (PCR) analysis of biopsies: ear for adult animals or cortical brain fragments for pups. The primers were: CGG GCC TCT TCG CTA TTA C (Chr3 Mutant Reverse), ACC CCC ATG TCA GAG TTC CT (Chr3 Common), TAT ACA ACC TTG GGG GAT GG (Chr3 WT Reverse). PCR from hemizygous animals produced two bands. Heterozygous and wild-type yielded a single band at the size of 129 and 216 bp, respectively. Homozygotes were excluded from the experiments.

### 4.2. Cell Cultures 

Cell cultures were maintained at 37 °C with 5% CO_2_ in a humidified incubator. Human embryonic kidney 293T (HEK293T) cells were cultured in Dulbecco’s Modified Eagle Medium (DMEM) with 10% fetal bovine serum (FBS) and penicillin/streptomycin. Mouse primary cultures of hippocampal neurons were prepared as described earlier [71]. Shortly, the hippocampi of newborn mice were dissected, digested with trypsin, and seeded onto poly-L-lysine treated coverslips or Petri’s dishes in Neurobasal-A medium with 3% FBS and 3% B27 supplement. We took a maximum of 8 pups from one litter for parallel hippocampal cultures. Tissues of a pup yielded one hippocampal culture in a Petri dish with 10–14 small (4 mm × 4 mm) coverslips for calcium imaging or 4–5 coverslips for immunostaining. If required, coverslips were split to different dishes for lentiviral infections or drug treatments. Experiments were performed on in vitro days 10–13. 

### 4.3. Lentiviral Infection

Some cultures were infected with lentiviruses five days before experiments. Lentiviruses were generated by transfecting HEK293T with polyethyleneimine and a mixture of three plasmids: envelope vector pMD2.G, packaging vector psPAX2 (AddGene #12259 and #12260 were gifts from Didier Trono), pGFP-C-shLenti vector (OriGene, Rockville, MD, USA, TR30021, referred to as mock shRNA), shRNA STIM1 or shRNA STIM2 vectors (a kind gift from prof. I. Bezprozvanny, Southwestern. Medical Center, Dallas, TX, USA). The supernatant with viruses was harvested 48 and 72 h after transfection, filtered, and concentrated by centrifugation (47,000 *g*, 2 h, 4 °C). Pellets were resuspended in NeuroBasal-A, aliquoted, and stored at −80 °C. Transduction efficacy was confirmed by western blotting and immunostaining of primary hippocampal neuron cultures of C3Ha mice.

### 4.4. Western Blotting

Total protein lysates were separated by 8–10% SDS-PAGE and transferred onto the nitrocellulose membrane. Membranes were blocked with 5–10% bovine serum albumin (BSA) and probed with antibodies. Antibodies raised against α-tubulin (Sigma, St. Louis, MO, USA, T9026, 1:1000), STIM1 (Cell Signaling, Danvers, MA, USA, №5668, 1:1000), STIM2 (Cell Signaling №4917, 1:1000), Orai1 (Sigma SAB4200273, 1:500), Orai2 (ProSci, Poway, CA, USA, №4111, 1:300), TRPC1 (Alomone, Jerusalem, Israel, ACC-010, 1:200) were used as primary antibodies; anti-mouse and anti-rabbit horseradish peroxidase-labeled antibodies were used as secondary antibodies (Sigma A0168 and A0545, 1:30,000). Signal was detected with SuperSignal West Femto Maximum Sensitivity Substrates and imaged with ChemiDoc Imaging System. Densitometry values were determined using Image Lab software. Target protein values were normalized to α-tubulin values. 

### 4.5. Immunostaining 

The modified AbCam protocol for immunostaining (www.abcam.com, accessed on 22 July 2019) was used. Cells on coverslips were fixed with 4% formaldehyde for 10 min, permeabilized with 0.25% TritonX-100 for 10 min, blocked with 10% FBS and 0.3M glycine in PBST (PBS with 0.1% Tween20) for 1 h, and subsequently incubated with antibodies in 1% BSA in PBST for 1 h. Primary antigen-specific antibodies included: STIM1 (Cell Signaling №5668, 1:800), STIM2 (Cell Signaling №4917, 1:50), and neuron-specific β-tubulin III (Sigma T8578, 1:500). Secondary antibodies were Alexa Fluor 555 anti-rabbit (Thermo Fisher Scientific, Waltham, USA, A-21428, 1:500) and Alexa Fluor 488 anti-mouse (Thermo Fisher Scientific A-11001, 1:2000). Nuclei were counterstained with DAPI in the mounting medium. Images were obtained with an IX83 microscope and Olympus FV3000 confocal laser system using an Olympus 40x objective (oil, 1.3 NA); the pinhole was one Airy disk. Fluorescence was excited by 405, 488, and 561 nm laser lines and detected. Representative z-stack images were captured using Olympus FluoView software (v2.1.1.98) and analyzed with FiJi software (v1.53f51) [72]. 

### 4.6. Calcium Imaging

Cells were loaded with 5 μM Fura-2 AM in 0.2 mM probenecid) in artificial cerebrospinal fluid (aCSF) for 55 min at 37 °C. aCSF contained (in mM): 140 NaCl, 5 KCl, 2 CaCl_2_, 1 MgCl_2_, 10 HEPES, 10 glucose; pH 7.3. The cytosolic Ca^2+^ concentration change was determined as the ratio of Fura-2 fluorescence intensities at 340 and 380 nm excitation wavelengths (F340/F380) with emission at 510 nm. Cells were imaged by an Andor’s Zyla 4.2 sCMOS camera driven by MicroManager Software [73]. Images were analyzed with FiJi [72]. F340/F380 curves are represented as the means with a standard error of the mean (SEM). Resting calcium levels were measured in 2 mM Ca^2+^ aCSF solution before any treatment. Calcium entry was calculated as the F340/F380 peak of each trace, and the baseline was subtracted (ΔF340/F380). Before internal calcium store depletion, cells were kept in Ca^2+^-free aCSF for 30 sec. Internal calcium stores were depleted in Ca^2+^-free aCSF solution with an addition of 1 μM thapsigargin (Tg; Sigma, T9033), or 5 μM ionomycin (Ion; Sigma I0634) or 25 μM caffeine (Sigma, C0750), supplemented with 1 μM nifedipine (Sigma, N7634) and 1 μM tetrodotoxin (TTX, Tocris, 1069). SOC entry was measured after Ca^2+^-readdition. Ca^2+^-free aCSF had 100 nM of free Ca^2+^ (MaxChelator calculation) and contained (in mM) 47.4 nM CaCl2 and 100 μM EGTA in aCSF. Some cells were pretreated for 20 h with 1 μM teriflunomide (Sigma SML0936) or leflunomide (Sigma, L5025). VGC entry was activated with a depolarizing solution containing (in mM) 50 KCl and 97.5 NaCl in aCSF (50 KCl aCSF). One mM of TPEN (Sigma, P4413) chelator in Ca^2+^-free aCSF was used to mimic store depletion. Calcium entry through L-type and T-type VGC channels was blocked by the application of 10 μM nifedipine or 10 μM ML218 (Sigma, SML0385) 1 min before depolarization with 50 KCl. A calcium imaging experiment yielded 3 to 30 individual neuron records from one coverslip. All the experiments were done blind to the genotype, and genotyping was done after gathering the data. Therefore, all the data sets represent all the data points obtained based on the genotyping results. No data points were removed. At least 3 animals per group were used in data sets.

### 4.7. Statistics

Statistical analysis was performed with OriginPro8.1 and Python. We analyzed datasets with estimation plots [74]. Estimation plots were created with a package for Data Analysis using Bootstrap-Coupled ESTimation (DABEST). Each estimation plot contains a scatter plot with raw data normalized by the control group and descriptive statistics for mean differences with effect sizes, sampling distributions, 95% confidence intervals (the vertical error bars). A total of 5000 bootstrap samples were taken from each group to draw sampling distributions. The Shapiro–Wilk test was applied to determine the data distribution to choose between parametric or non-parametric tests for further analysis. Unpaired and paired *t*-tests were applied for the comparison of Western blotting and immunofluorescence data. The Mann–Whitney test or non-parametric ANOVA (Kruskal–Wallis test) with post hoc Dunn’s test with Bonferroni correction was applied for calcium data analysis. Both Mann–Whitney and Dunn’s were used as non-parametric tests that do not assume normal distributions. Dunn’s test was used for non-parametric pairwise multiple comparisons [75]. Fisher’s exact test was used to assess frequency difference. *p* values below 0.05 were considered significant. Mean, SEM, and statistical data are presented in Supplementary Tables S1–S14. 

## Data Availability

The raw and analyzed datasets are available on request to the corresponding author.

## Supplementary Materials

The following supporting information can be downloaded at: https://www.mdpi.com/article/10.3390/ijms232314810/s1.
