# Supplementary material for: The STIM1/2-Regulated Calcium Homeostasis Is Impaired in Hippocampal Neurons of the 5xFAD Mouse Model of Alzheimer’s Disease
Source: Int J Mol Sci. 2022 Nov 26;23(23):14810. doi: 10.3390/ijms232314810 (PMC9738900; doi:10.3390/ijms232314810)
Supplement: Supplementary file 1 [file ijms-23-14810-s001.zip › Skobeleva Suppl Figure_UPDATED.pdf]

Supplement Figure S1

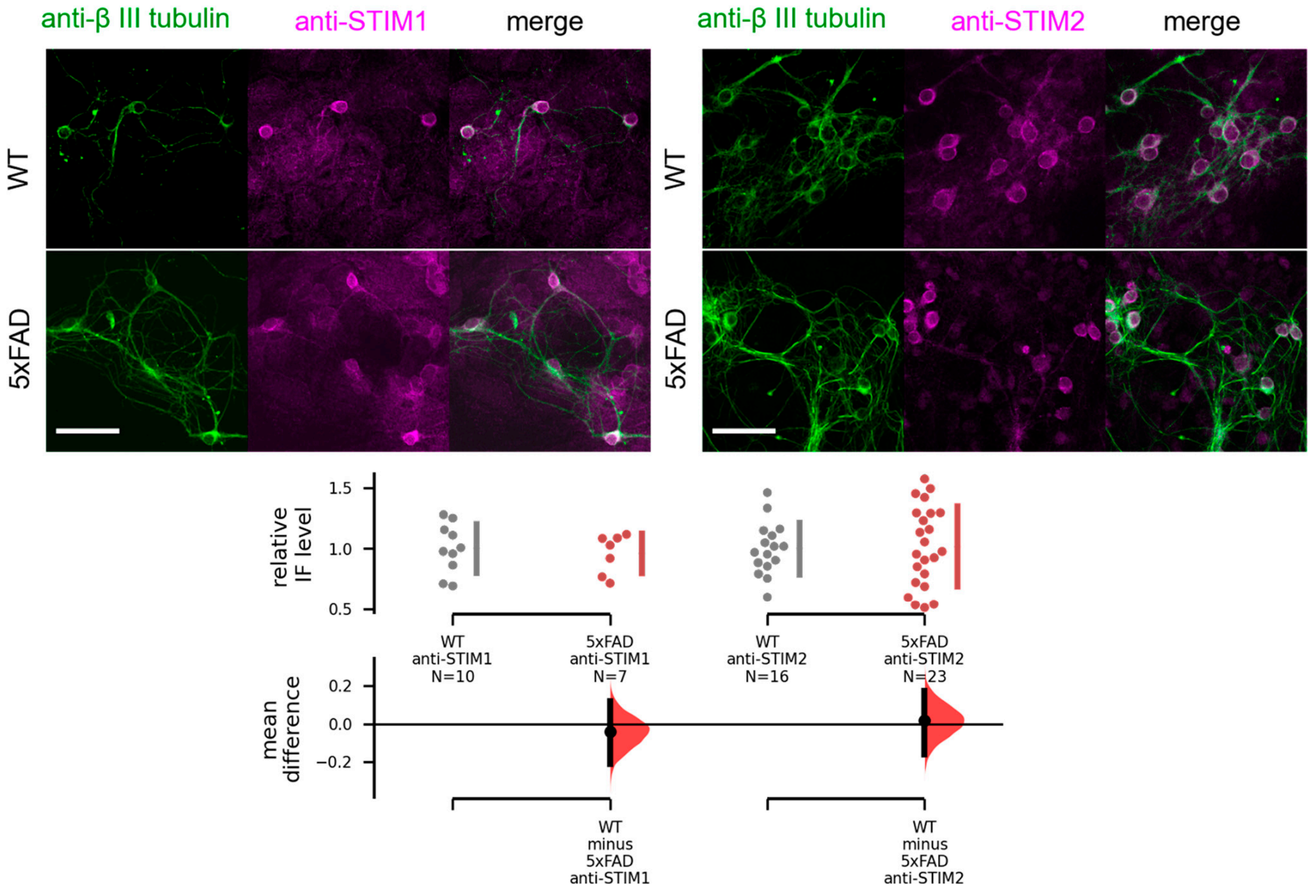

Confocal immunofluorescence images of neuronal cultures of wild-type (WT) and 5xFAD mice probed with anti-STIM1/2 (magenta, anti-STIM1 and anti-STIM2) and β-III neuro-tubulin antibodies (green, anti-β III neuro-tubulin). Scale bar is 50µm. Integrated immunofluorescence for anti-STIM1/2 staining was calculated for every neuron counterstained with anti-β III neuro-tubulin. Datasets were normalized to WT values to determine relative immunofluorescence (IF) levels. Normalized data are plotted on the upper axes; mean differences are plotted on floating axes on the bottom as a bootstrap sampling distribution. We found no significant differences between WT and 5xFAD cultures: mean differences were -0.04 ($p = 0.67$, t-test) for anti-STIM1 staining and 0.017 ($p = 0.86$, t-test) for anti-STIM2 staining.

Supplement Table S1. Statistical values and statistics for datasets in Figure 1-5. "diff" is short for difference.

Type of Data	Mean±SEM	N	Statistics
Figure 1 A *Normalized SOC entry values*			
WT	1.00±0.07	79	p = 0.000002 (Mann-Whitney test) difference (diff) = 0.42 (+42%)
5xFAD	1.42±0.05	156	
Figure 1 B *Normalized entry values after TPEN treatment*			
WT	1.00±0.09	21	p = 4.18e-07 (Mann-Whitney test) diff = 1.31 (+131%)
5xFAD	2.31±0.22	26	
Figure 1 C *Normalized SOC entry values for WT or 5xFAD neurons treated with leflunomide (Lef), teriflunomide (Ter), BTP2 or DMSO*			
WT + DMSO	1.00±0.03	162	KW ANOVA p = 5.83e-10, Dunn's test p values in Suppl. Table S2
WT + Lef	0.74±0.04	54	diff = -0.25 (-25%) comp. with WT DMSO
WT + Ter	1.04±0.05	55	diff = 0.04 (4%) comp with WT DMSO
5xFAD + DMSO	1.22±0.08	53	
5xFAD + Lef	0.83±0.05	64	diff = -0.40 (-32%) comp. with 5xFAD DMSO
5xFAD + Ter	0.85±0.05	40	diff = -0.37 (-30%) comp. with 5xFAD DMSO
5xFAD + BTP2	0.66±0.06	31	diff = -0.57 (-47%) comp. with 5xFAD DMSO
Figure 2A *Relative expression levels calculated as densitometry of values for the target protein and normalized to α-tubulin value (Western Blotting)*			
WT STIM1	1.00±0.07	15	diff = -0.03 p = 0.74 (t-test)
5xFAD STIM1	0.97±0.07	14	
WT STIM2	1.00±0.10	8	diff = -0.04 p = 0.9 (t-test)
5xFAD STIM2	0.96±0.28	6	
WT Orai1	1.00±0.13	6	diff = 0.2 p = 0.67 (t-test)
5xFAD Orai1	1.20±0.52	4	
WT Orai2	1.00±0.08	5	diff = 0.05 p = 0.8 (t-test)
5xFAD Orai2	1.05±0.21	4	
WT TRPC1	1.00±0.17	6	diff = -0.21 p = 0.38 (t-test)
5xFAD TRPC1	0.79±0.12	4	
Figure 2B *Raw resting calcium levels were measured as F340/F380*			
WT	0.36±0.004	185	diff = 0.006 P = 0.75 (Mann-Whitney test)
5xFAD	0.37±0.003	252	
Figure 2C *The normalized Ca2±release **peaks** of WT and 5xFAD neurons in response to thapsigargin (Tg)*			
WT	1.0±0 2	28	diff = -0.24 p = 0.04 (Mann-Whitney test)

5xFAD	0.76±0 2	95	
Figure 2C *The normalized Ca2±release* ***AUC values*** *of WT and 5xFAD neurons in response to thapsigargin (Tg)*			
WT	1.00± 0.14	28	diff = -0.25<br>p = 0.03 (Mann-Whitney test)
5xFAD	0.88± 0.07	95	
Figure 2D *The normalized Ca2±release* ***peaks*** *of WT and 5xFAD neurons in response to ionomycin (Ion)*			
WT	1.0±0.09	39	diff = 0.39<br>p = 0.009 (Mann-Whitney test)
5xFAD	1.39 ± 0.1	70	
Figure 2D *The normalized Ca2±release* ***AUC values*** *of WT and 5xFAD neurons in response to ionomycin (Ion)*			
WT	1.0 ± 0.13	39	diff = 0.016<br>p = 0.84 (Mann-Whitney test)
5xFAD	1.02 ± 0.1	70	
Figure 2E *The normalized Ca2±release* ***peaks*** *of WT and 5xFAD neurons in response to caffeine (Caff)*			
WT	1.00±0.31	14	diff = 0.22<br>p = 0.28 (Mann-Whitney test)
5xFAD	1.22±0.17	32	
Figure 2E *The normalized Ca2±release* ***AUC values*** *of WT and 5xFAD neurons in response to caffeine (Caff)*			
WT	1.00±0.11	14	diff = -0.24<br>p = 0.84 (Mann-Whitney test)
5xFAD	0.76± 0.11	32	
**Figure 3A** *Normalized SOC entry values for WT and 5xFAD neurons with STIM1/STIM2 KD*			
WT	1.0 ± 0.07	79	KW ANOVA p = 2.14e-22<br>Dunn'test p values in Suppl. Table S3
WT STIM1 KD	1.15 ± 0.1	51	diff = -0.15 (-15%) comp with WT
WT STIM2 KD	0.48 ± 0.08	33	diff = -0.52 (-52%) comp with WT
5xFAD	1.42 ± 0.05	156	
5xFAD STIM1 KD	0.49 ± 0.09	15	diff = -0.94 (-66%) comp with 5xFAD
5xFAD STIM2 KD	0.82 ± 0.09	62	diff = -0.60 (-42%) comp with 5xFAD
Figure 3B *Relative expression levels of STIM1/2 normalized to α-tubulin value (Western blotting)*			
WT anti-STIM1	1	9	diff = -0.39 (-39%) p = 0.001 (paired t-test)
5xFAD anti-STIM1	0.61±0.08	9	
WT anti-STIM2	1	6	diff = -0.44 (-44%) p = 0.003 (paired

			t-test)
5xFAD anti-STIM2	0.56±0.09	6	
Figure 3C *Normalized Integrated immunofluorescence for anti-STIM1/2 staining*			
C3HA neurons Mock anti-STIM1	1.0±0.02	38	diff = -0.51 (-51%) p = 1.40e-32 (t-test)
C3HA neurons STIM1 KD anti-STIM1	0.49±0.01	41	
C3HA neurons Mock anti-STIM2	1.02±0.11	11	diff = -0.57 (-57%) p = 5.49e-05 (t-test)
C3HA neurons STIM2 KD anti-STIM2	0.45±0.06	15	
Figure 4A *Normalized depolarization-induced Ca2+ entry **peak** values*			
WT	1.0±0.04	106	diff = 0.19 (+19%) p = 1.6E-4 (Mann-Whitney test)
5xFAD	1.19±0.04	96	
Figure 4A *Normalized depolarization-induced Ca2+ entry **AUC** values*			
WT	1.0±0.04	87	diff = 0.17 (+17%) p = 0.002 (Mann-Whitney test)
5xFAD	1.17±0.04	97	
Figure 4B *Normalized depolarization-induced Ca2+ entry **peak** values after TPEN treatment*			
WT after TPEN	1.00±0.08	38	diff = 0.013 p = 0.98 (Mann-Whitney test)
5xFAD after TPEN	1.01±0.04	133	
Figure 4B *Normalized depolarization-induced Ca2+ entry **AUC** values after TPEN treatment*			
WT after TPEN	1±0.07	37	diff = 0.28 p = 0.006 (Mann-Whitney test)
5xFAD after TPEN	1.28±0.05	133	
Figure 4B *Normalized depolarization-induced Ca2+ entry **AUC** values after TPEN treatment compared with WT and 5xFAD with replete stores (separate recording)*			
WT	1.00±0.04	101	KW ANOVA p =7.70e-44 Dunn's test values in Suppl. Table S4
5xFAD	1.60 ±0.03	288	
WT after TPEN	0.73 ± 0.04	37	
5xFAD after TPEN	0.93 ± 0.04	133	
Figure 4C *Normalized depolarization-induced Ca2+ entry **peak** values in neurons treated with inhibitors*			
5xFAD	1.16 ± 0.06	33	KW ANOVA p = 1.28-05 Dunn's test values in Suppl. Table S5
5xFAD Nif	0.73±0.06	24	
5xFAD ML 218	0.85±0.04	28	
Figure 4C *Normalized depolarization-induced Ca2+ entry **AUC** values in neurons treated with inhibitors*			
5xFAD	1.13 ± 0.05	33	KW ANOVA p = 3.94-09 Dunn's test values in Suppl. Table S6
5xFAD Nif	0.55±0.05	24	
5xFAD ML 218	0.93±0.04	28	
Figure 5A *Normalized depolarization-induced Ca2+ entry **peak** values in neurons with STIM1/2 KD*			
WT	1.0±0.04	106	KW ANOVA p = 0.007

WT STIM1 KD	1.09 ± 0.08	29	Dunn’s test values in Suppl. Table S7
WT STIM2 KD	1.29 ± 0.08	38	
Figure 5A<br>*Normalized depolarization-induced Ca2+ entry **AUC** values in neurons with STIM1/2 KD*			
WT	1.0±0.04	87	KW ANOVA p = 7.67 E-5<br>All pairwise Dunn’s test p values = 1
WT STIM1 KD	0.93 ± 0.06	29	
WT STIM2 KD	1.06 ± 0.11	38	
Figure 5B<br>*Normalized depolarization-induced Ca2+ entry **peak** values in neurons with STIM1/2 KD*			
5xFAD	1.19±0.04	96	KW ANOVA p = 1.28e-05<br>Dunn’s test values in Suppl. Table S8
5xFAD STIM1 KD	1.28 ± 0.08	21	
5xFAD STIM2 KD	1.38±0.05	63	
Figure 5B<br>*Normalized depolarization-induced Ca2+ entry **AUC** values in neurons with STIM1/2 KD*			
5xFAD	1.17±0.04	97	KW ANOVA p =3.94e-09<br>Dunn’s test values in Suppl. Table S9
5xFAD STIM1 KD	1.35 ± 0.1	21	
5xFAD STIM2 KD	1.44 ± 0.05	63	
Figure 5C<br>*Normalized depolarization-induced Ca2+ entry **peak** values in neurons with STIM1 KD treated with inhibitors*			
5xFAD STIM1 KD	1.28 ± 0.08	21	KW ANOVA p = 7.85e-07<br>Dunn’s test values in Suppl. Table S10
5xFAD STIM1 KD + NIF	0.76 ± 0.05	33	
5xFAD STIM1 KD + ML	0.68 ± 0.04	20	
Figure 5C<br>*Normalized depolarization-induced Ca2+ entry **AUC** values in neurons with STIM1 KD treated with inhibitors*			
5xFAD STIM1 KD	1.35 ± 0.1	21	KW ANOVA p = 5.85e-09<br>Dunn’s test values in Suppl. Table S11
5xFAD STIM1 KD + NIF	0.62 ± 0.03	33	
5xFAD STIM1 KD + ML	0.68 ± 0.03	20	
Figure 5D<br>*Normalized depolarization-induced Ca2+ entry **peak** values in neurons with STIM2 KD treated with inhibitors*			
5xFAD STIM2 KD	1.38±0.05	63	KW ANOVA p = 0.0032<br>Dunn’s test values in Suppl. Table S12
5xFAD STIM2 KD + NIF	0.92 ± 0.05	9	
5xFAD STIM2 KD + ML	1.30 ± 0.09	20	
Figure 5D<br>*Normalized depolarization-induced Ca2+ entry **AUC** values in neurons with STIM2 KD treated with inhibitors*			
5xFAD STIM2 KD	1.44 ± 0.05	63	KW ANOVA p = 5.19E-05<br>Dunn’s test values in Suppl. Table S13
5xFAD STIM2 KD + NIF	0.82 ± 0.04	9	
5xFAD STIM2 KD + ML	1.2 ± 0.08	20	

Supplement Table S2. Dunn’s test pair wise comparison for normalized SOC entry values for neurons with FDA-approved drugs (Figure 1C)

	WT + DMSO	WT + Lef	WT + Ter	5xFAD + DMSO	5xFAD + Lef	5xFAD + Ter	5xFAD + BTP2

WT + DMSO	1	0.002	1	0.67	0.027	1.00	0.0017
WT + Lef	0.002	1	0.0004	0.000013	1.00	1.00	1
WT + Ter	1	0.0004	1	1	0.005	0.50	0.00032
5xFAD + DMSO	0.67	0.00001	1	1	0.00024	0.07	0.000018
5xFAD + Lef	0.027	1	0.005	0.00024	1	1	1
5xFAD + Ter	1	1	0.50	0.07	1	1	0.76
5xFAD + BTP2	0.0017	1	0.00032	0.00	1	0.76	1

Supplement Table S3. Dunn's test pair wise comparison for normalized SOC in neurons with STIM1 or STIM2 knock down (KD) (Figure 1C)

	WT	WT STIM1 KD	WT STIM2 KD	5xFAD	5xFAD STIM1 KD	5xFAD STIM2 KD
WT	1	1	2.31E-04	2.87E-05	0.026	0.36
WT STIM1 KD	1	1	1.59E-05	0.06	0.004	0.034
WT STIM2 KD	2.31E-04	1.60E-05	1	7.71E-15	1	0.25773
5xFAD	2.90E-05	0.06	0	1	1.78E-07	0
5xFAD STIM1 KD	0.026	0.0036	1	1.78E-07	1	1
5xFAD STIM2 KD	0.36	0.034	0.25773	0	1	1

Supplement Table S4 Dunn's test pair wise comparison for normalized **AUC** values KCl responses for WT and 5xFAD neurons with replete and depleted (after TPEN treatment) calcium stores

	WT	WT after TPEN	5xFAD	5xFAD after TPEN
WT	1	0.036	1.42E-17	1
WT after TPEN	0.036	1	7.74E-18	0.15
5xFAD	1.42E-17	7.74E-18	1	7.79E-26
5xFAD after TPEN	1	0.15	7.79E-26	1

Supplement Table S5. Dunn's test pair wise comparison for normalized **peak** values for 5xFAD neurons treated with nifedipine (Nif) an d ML 218 (ML) (Figure 4C)

	5xFAD + DMSO	5xFAD + Nif	5xFAD + ML

5xFAD + DMSO	1	3.20E-05	1
5xFAD + Nif	3.20E-05	1	2.53E-04
5xFAD + ML	1	2.53E-04	1

Supplement Table S6. Dunn's test pair wise comparison for normalized **AUC** values for 5xFAD neurons treated with nifedipine (Nif) an d ML 218 (ML) (Figure 4C)

	5xFAD + DMSO	5xFAD + Nif	5xFAD + ML
5xFAD + DMSO	1.00	1.85E-09	0.10
5xFAD + Nif	1.85E-09	1.00	1.86E-04
5xFAD + ML	0.10	1.86E-04	1.00

Supplement Table S7. Dunn's test pair wise comparison for normalized **peak** values for WT neurons treated with STIM1 or STIM2 KD (Figure 5A)

	WT	WT STIM1	WT STIM2
WT	1	0.92	0.005
WT STIM1	0.92	1	0.38
WT STIM2	0.005	0.38	1

Supplement Table S8. Dunn's test pair wise comparison for normalized **peak** values for 5xFAD neurons with STIM1 KD (Figure 5B)

	5xFAD	5xFAD STIM1 KD	5xFAD STIM2 KD
5xFAD	1	0.78	0.005
5xFAD STIM1 KD	0.78	1	1
5xFAD STIM2 KD	0.005	1	1

Supplement Table S9. Dunn's test pair wise comparison for normalized **AUC** values for 5xFAD neurons with STIM1 KD (Figure 5B)

	5xFAD	5xFAD STIM1 KD	5xFAD STIM2 KD
5xFAD	1	0.27	4.80E-05
5xFAD STIM1 KD	0.27	1	0.75
5xFAD STIM2 KD	4.80E-05	0.75	1

Supplement Table S10. Dunn's test pair wise comparison for normalized **peak** values for 5xFAD neurons with STIM1 KD (5xFAD STIM1 KD) treated with nifedipine (Nif) an d ML 218 (ML) (Figure 5C)

	5xFAD STIM1 KD	5xFAD STIM1 KD + Nif	5xFAD STIM1 KD + ML
5xFAD STIM1 KD	1	4.40E-05	3.00E-06
5xFAD STIM1 KD + Nif	4.40E-05	1	0.74
5xFAD STIM1 KD + ML	3.00E-06	0.74	1

Supplement Table S11. Dunn's test pair wise comparison for normalized **AUC** values for 5xFAD neurons with STIM1 KD (5xFAD STIM1 KD) treated with nifedipine (Nif) an d ML 218 (ML) (Figure 5C)

	5xFAD STIM1 KD	5xFAD STIM1 KD + Nif	5xFAD STIM1 KD + ML
5xFAD STIM1 KD	1	6.63E-09	2.10E-05
5xFAD STIM1 KD + Nif	6.63E-09	1	1
5xFAD STIM1 KD + ML	2.14E-05	1	1

Supplement Table S12. Dunn's test pair wise comparison for normalized **peak** values for 5xFAD neurons with STIM2 KD (5xFAD STIM2 KD) treated with nifedipine (Nif) an d ML 218 (ML) (Figure 5D)

	5xFAD STIM2 KD	5xFAD STIM2 KD + Nif	5xFAD STIM2 KD + ML
5xFAD STIM2 KD	1	0.0022	1
5xFAD STIM2 KD + Nif	0.0022	1	0.037
5xFAD STIM2 + ML	1	0.037	1

Supplement Table S13. Dunn's test pair wise comparison for normalized **AUC** values for 5xFAD neurons with STIM2 KD (5xFAD STIM2 KD) treated with nifedipine (Nif) an d ML 218 (ML) (Figure 5D)

	5xFAD STIM2 KD	5xFAD STIM2 KD + Nif	5xFAD STIM2 KD + ML
5xFAD STIM2 KD	1	8.20E-05	0.09
5xFAD STIM2 KD + Nif	8.20E-05	1	0.06
5xFAD STIM2 KD + ML	0.09	0.06	1

Supplement Table S14. Statistics for Supplement Figure 1.

**Type of Data**	**Mean±SEM**	**N**	**Statistics**
WT anti-STIM1	1.0±0.06	10	diff = -0.04 (-4%)<br>p = 0.67 (t-test)
5xFAD anti-STIM1	0.96±0.06	7	

WT anti-STIM2	1.0±0.05	16	diff = 0.016<br>p = 0.86 (t-test)
5xFAD anti-STIM2	1.02±0.07	23	
